# The Pros and Cons of Low Carbohydrate and Ketogenic Diets in the Prevention and Treatment of Cancer

**DOI:** 10.3389/fnut.2021.634845

**Published:** 2021-02-25

**Authors:** Ingrid Elisia, Gerald Krystal

**Affiliations:** The Terry Fox Laboratory, BC Cancer, Vancouver, BC, Canada

**Keywords:** ketogenic, low carbohydrate, cancer, safety, prevention, treatment, inflammation

## Abstract

Ketogenic diets are low carbohydrate (CHO), high fat diets that are currently very popular for weight loss. Since cancer cells typically consume far more glucose than normal cells, low CHO diets are currently being considered as possible therapeutic regimens to manage cancer. However, our understanding of the safety and efficacy of such CHO-restricted diets in the prevention and treatment of cancer is still in its infancy. In this perspective we provide an overview of the current state of knowledge regarding the use of low CHO diets in the prevention and treatment of cancer. We also highlight the gaps in our knowledge regarding the potential usefulness of low CHO diets in cancer. While pre-clinical rodent studies have provided convincing evidence that CHO restriction may be effective in reducing cancer growth, there has not been sufficient attention given to the effect of these low CHO diets, that are often high in fats and low in soluble fiber, on inflammation. This is important, given that different fats have distinct effects on inflammation. As well, we demonstrate that short chain fatty acids, which are produced via the fermentation of fiber by our gut microbiome, have more anti-inflammatory properties than β-hydroxybutyrate, a ketone body produced during nutritional ketosis that is touted to have anti-inflammatory activity. Since chronic inflammation is strongly associated with cancer formation, defining the type of fats in low CHO diets may contribute to our understanding of whether these diets may work simply by reducing glucose bioavailability, or via modulation of inflammatory responses.

## Introduction

Low carbohydrate (CHO) diets restrict CHO intake while increasing fat and/or protein. There are currently many low CHO diets, including ketogenic diets, and they vary in their stringency of CHO restriction and the amount as well as type of fat and protein (1, 2). Historically, the ketogenic diet was first used in the 1920s to treat pediatric epilepsy (3). The use of CHO restriction for weight loss was popularized a couple of decades later with the introduction of the Atkins diet (4). Today, ketogenic diets are immensely popular as a weight loss regimen (5).

In this perspective, we provide an overview of the current state of knowledge regarding the safety and efficacy of low CHO diets for cancer prevention and treatment. In addition, we propose further studies to bring CHO restriction therapy into the clinic, specifically for cancer.

## Carbohydrate Restriction and Cancer

In the 1920s, Otto Warburg observed that cancer cells take up more glucose than normal cells and convert it via glycolysis to lactic acid (6, 7). This characteristic of cancer cells has been called the Warburg effect or aerobic glycolysis (since this increased glycolysis occurs even when adequate oxygen is present) (6). This was an unexpected finding since glycolysis is inefficient at producing ATP, generating only 2 ATPs/glucose, and cancer cells typically need lots of energy for their uncontrolled proliferation (6). As well, it was thought at that time that cells only relied on glycolysis under anaerobic conditions, since the downstream citric acid cycle, which converts pyruvate to carbon dioxide and generates far more ATP than glycolysis, requires oxygen to operate (6, 7). However, cancer cells, and all rapidly growing cells, prefer increasing glycolysis over increasing the citric acid cycle since it generates both reduced glutathione to combat oxidative stress (which is increased in cancer cells and can kill them) and biosynthetic precursors required to support cell proliferation (6). Since cancer cells take up and need more glucose than normal cells, it is therefore reasonable to hypothesize that a reduction in CHO intake, which increases glucose in the blood, might help prevent or treat cancer.

Another possible mechanism by which CHO restriction, and specifically a ketogenic diet, might slow cancer growth is through the generation of ketone bodies, since there is some evidence that many cancer cells, unlike normal cells, cannot use ketone bodies as an energy source (8–10). Ketone bodies are generated via fatty acid oxidation in the liver when blood glucose levels drop. They then travel in the blood to all our cells and are converted into acetyl-CoA by β-OHB dehydrogenase1 (BDH1), succinyl-CoA:3-oxoacid-CoA transferase (SCOT) and acetoacetyl-CoA thiolase and enter the citric acid cycle to generate ATP (11, 12). However, while many cancer cells do not appear to have the enzymes required to break down ketone bodies to acetyl-CoA, and are therefore at a selective disadvantage in a low glucose environment (8–10, 13), some cancer cell types do (10) so a ketogenic diet may not be effective at preventing or treating all cancers.

## The Safety of Low Carbohydrate Diets

Adoption of low CHO diets in humans has received pushback from the scientific community because a reduction in CHO is often compensated by an increase in fat, which typically includes saturated fats. This is often the main criticism of low CHO, high fat diets, i.e., the consumption of high amounts of saturated fat may lead to increased levels of LDL cholesterol (14, 15), which in turn raises cardiovascular risk (16, 17). There are now, however, many clinical trials demonstrating that consumption of saturated fats in the context of low CHO, high fat diets, while increasing LDL cholesterol, tends to reduce plasma triglycerides and increase HDL cholesterol (14, 18, 19). In keeping with this, a recent meta-analysis reported no association between intake of saturated fats and all cause mortality, cardiovascular disease, and coronary heart disease mortality. However, an association with trans fats was reported (20). Furthermore, low CHO, high fat diets appear to reduce small, dense LDL cholesterol (21, 22), which is thought to be the type of cholesterol associated with coronary artery disease (17, 23). However, these clinical trials are relatively short term and there is still a lack of long-term studies evaluating the effect of low CHO, high fat diets on cardiovascular disease.

## Low Carbohydrate Diets in Pre-clinical Studies

The potential efficacy of low CHO or ketogenic diets to prevent or treat cancer has been obtained primarily from rodent studies. Many of these studies have employed either human cancer cells in immunocompromised mice (xenograft models), or implanted murine tumor cells in syngeneic mice (24). Using these model systems, we and others have demonstrated that low CHO diets exhibit anti-tumor activity against colon (25–28), gastric (29), prostate (30, 31), head and neck (26), brain (32–35), and thyroid (35) cancer. A more extensive list of publications reporting the efficacy of CHO restrictive diets or ketogenic diets in slowing tumor growth in rodent model systems can be found in a recent review by Weber et al. (24).

Despite these promising results, there are other studies demonstrating that low CHO, high fat diets promote cancer progression (36). For example, Zhang et al. (10) have demonstrated that specific cancer cells possess the ketolytic enzymes required to break down ketone bodies to acetyl Co-A, and this in turn can feed into the citric acid cycle and be used to generate ATP. It is thus possible that the efficacy of a ketogenic diet will turn out to be dependent on whether the cancer cells express high or low levels of enzymes involved in converting ketone bodies to acetyl-CoA.

Results from our lab have demonstrated that a low CHO, high protein diet effectively lowers blood glucose and insulin levels and this is paralleled by a reduction in the growth of subcutaneously implanted murine and human cancer cells (26). We also showed that the same diet reduced tumor penetrance in a transgenic mouse model of HER-2/Neu–induced mammary cancer, while extending the lifespan of these mice (26). When combined with celecoxib, a cyclooxygenase 2 (COX2) inhibitor, this low CHO, high protein diet not only reduced lung metastasis of 4T1 mammary tumors in Balb/C mice, but also lowered the incidence of metastasis in the male Transgenic Adenocarcinoma of the Mouse Prostate (TRAMP) mice (37). This is despite not observing a significant change in LNCaP xenograft tumor growth (38). In castrated mice, however, our low CHO, high protein diet was effective in slowing tumor growth (38).

While xenografts or implanted tumor models are valuable at identifying agents that can slow the growth of established tumors, they cannot evaluate effects on tumor incidence. As well, xenografts typically rely on immunocompromised mice, which rules out the possible interaction between diets and the immune system, a relationship that we now appreciate plays an important role in cancer development (39). In addition, most cancers arise from somatic mutations that occur over a long period of time, and not from genetic mutations or from implanted tumors, which the above models represent (40).

To determine if CHO restriction might have an impact on cancer initiation and progression in a longer-term model, we tested the effect of a low CHO diet in preventing the formation of cigarette smoke carcinogen (NNK)-induced lung cancer in A/J mice (41). Specifically, we gave these A/J mice a Western diet or a variety of low CHO, high fat diets prior to NNK injections, which are needed to initiate lung cancer formation. In these studies, we evaluated the effect of different CHO, protein and fat types. Specifically, we compared the effect of carbohydrate types, soy protein vs. casein, as well as a blend of fats typically found in a Western diet with ones enriched in fish oil or coconut oil. Comparing the effect of fat types in a low CHO, high fat diet in this study is a particularly important research question to address since there have been very few studies to date clarifying the contribution of different fat types on the efficacy of the diet. Fish oil is an important source of omega 3 fatty acids such as eicosapentaenoic acid (EPA) and docosahexaenoic acid (DHA), while coconut oil is a popular type of fat that is often consumed in a ketogenic diet because of its high level of medium chain fatty acids (42). Medium chain triglycerides (MCTs) are reported to be more readily utilized for fatty acid oxidation than long chain fatty acids (43). Thus, they are metabolized for ATP generation more readily than longer chain fatty acids (43).

From these studies we demonstrated that while CHO restriction alone was sufficient to reduce NNK-induced lung nodule formation, partial substitution of the fats typically consumed in a Western diet with fish oil produced a far more robust reduction in lung nodule formation (41). On the other hand, incorporation of coconut oil had no impact on lung nodule count. In addition, when soy protein was included in the low CHO, high fish oil diet instead of casein, there was a further suppression of lung cancer formation, suggesting the superiority of soy protein over this milk protein.

This low CHO diet containing soy protein and enriched in fish oil also proved effective in slowing tumor growth in the NNK-induced lung cancer model when given to mice after lung nodules were established (44). Interestingly, in these studies with already established tumors, mice fed with this low CHO diet had similar tumor stage progression and tumor cell proliferation rates to mice on a Western diet but increased cleaved caspase 3, suggesting that the diet-induced reduction in tumor size could be attributed, at least in part, to the ability of the diet to induce apoptosis in the tumors (44).

Taken together, our studies suggest that while lowering glucose bioavailability may alone be an effective strategy to prevent cancer formation, incorporation of soy protein and fish oil into such low CHO diets may dramatically improve the efficacy of these diets in reducing lung cancer risk.

## Effect of Low Carbohydrate Diets on Inflammation

It is currently clear that chronic inflammation is a risk factor for cancer (45). Low grade chronic inflammation that does not resolve may lead to DNA mutations that can trigger cancer formation. At the same time, tumor-promoting inflammation is a critical component of cancer progression and is now one of the hallmarks of cancer (46). Considering the important contribution of inflammation to cancer, consumption of a diet or dietary component(s) that may modulate inflammation may be beneficial in both cancer prevention and treatment.

From our studies, it is apparent that a reduction in blood glucose alone may not give a robust reduction in lung cancer risk (41). While all versions of the low CHO diets we tested were effective in lowering blood glucose levels, the low CHO diet containing soy protein and fish oil was not only the most effective at lowering lung nodule formation but also the most effective at reducing insulin and pro-inflammatory cytokine levels (IL-6, IL-1β, and TNF-α) (41, 44). This suggests multi-pronged mechanisms may be at play in preventing cancer formation. For example, since fish oil is high in omega 3 PUFAs (EPA/DHA), it is possible that the omega 3 fatty acids play a critical role in reducing chronic inflammation and this, in turn, reduces the risk of lung tumor formation (47).

One of the major concerns regarding a CHO restrictive diet is its potential to promote inflammation because of the large amount of fats, particularly saturated fats, typically consumed in such diets (48, 49). While different types of fats may elicit pro- or anti-inflammatory responses, saturated fat has been shown to mimic lipopolysaccharide (LPS), which induces inflammation upon binding to this receptor on the surface of macrophages/monocytes and other innate immune cells (48, 50). On the other hand, polyunsaturated fats such as the omega 3 fatty acids, EPA and DHA, have been shown to exhibit anti-inflammatory effects (48, 51). Currently, most diet studies do not report their fatty acid composition other than whether the fatty acids are saturated or unsaturated (Table 1). This is a serious omission since, as mentioned, EPA and DHA, have anti-inflammatory activities that are not observed with other polyunsaturated fatty acids such as the omega 6 fatty acid, arachidonic acid (79). It is also known that it takes months for tissue EPA/DHA levels to peak, which means that the full effect of these high fat diets may only be detected in long term studies (80).

**Table 1:** Effect of low carbohydrate diets on metabolic and inflammatory markers that may influence cancer risk.

**No of subjects**	**CHO intake**	**CHO: protein:** **fat (kcal)** **ratio**	**Saturated:** **monounsaturated:** **Polyunsaturated** **fats ratio**	**Type of fatty acids**	**Duration**	**Compared to**	**Reduced calorie intake**	**Peak BHB (mM)**	**Weight**	**Blood glucose**	**Insulin**	**CRP**	**IL6**	**TNF-α**	**Other inflammatory markers**	**References**
33 Obese with MetS	<20–40 g/d	4:35:61	40:46:14		12 mo	Baseline	Yes	~0.5	↓	↓	↓	↓				(18)
52 Overweight/ obese		4:35:61			8 wk	Baseline			↓	↓	↓	↓				(52)
10 Obese with NAFLD	23–30/d	4:24:72			2 wk	Baseline	Isocaloric	~2	=				↓	↓		(53)
51 Diabetic	20–25g/d	5:20:75			6 mo	Low fat diet			↓	=		=	=		Soluble E-selectin (↓), ICAM1 (↓)	(54)
35 Obese		5:20:75	33:22:7		12 wk	Baseline	Initial 2 weeks	~2	↓	=	↓	=				(55)
17 Healthy men		5:15:80			4 wk	Habitual diet	Yes	0.77	↓	0	↓	↑	=			(56)
19 Overweight		5:31:64	39:33:16		7 d	Baseline			↓	↓		=	=		MCP1 (↓)	(57)
9 Healthy	<50 g/d	7.5:27:65			4 wk	Habitual diet		0.7	↓	0			=			(58)
15 Overweight men		8:28:63	44:36:20		6 wk	Baseline	Yes		↓			↓	↓	↓	ICAM1 (↓)	(59)
15 Obese	<40 g/d	10:34:56	41:42:17		12 wk	Baseline	Yes		↓	↓	↓	↓		=		(60)
10 Healthy women		10:30:60	42:36:21		4 wk	Baseline						=	=	=		(14)
16 Diabetics		10:25:65	23:60:17		4 d	Baseline			↓	↓	=		=	=	MCP1 (=), pJNK (↓), IL10(=), IL18 (=)	(61)
21 Statin takers	<50 g/d	11:28:58	51:35:14		6 wk	Baseline	Yes	~0.5	↓	=	↓	=	=	=	IL8 (=), soluble E-selectin (↓), ICAM-1 (↓)	(62)
34 Overweight male		11:43:46		EPA/DHA	4 wk	No omega 3, ketogenic diet			=	=	↓		↓	↓	IL1β (↓)	(63)
						Baseline	Yes		↓	↓	↓		↓	↓	IL1β (↓)	
20 Overweight		12:29:59	48:35:16	Described	12 wk	Low fat diet	Yes		↓			↓	=	↓	VEGF (↓), P-selectin (↓), IL8 (↓), MCP1 (↓), E-Selectin (↓), PAI (↓), ICAM-1 (↓)	(64)
8 Men		12:30:58	30:44:26	Described	6 wk	Baseline	No	0.26	=	=	=	=	=	=	IL8 (=), MCP1 (=)	(65)
29 Overweight women		12:30:58			4 wk	Baseline	Yes		↓	=		↑	=			(66)
29 Overweight/obese men		13:27:60	37:45:18		12 wk	Baseline	Yes		↓			↓	=	↓		(67)
12 With metabolic syndrome	<50 g/d	16:22:62	45:40:15		4 wk	Baseline	Yes	0.53	↓	↓	↓	↓	↓	↓	ICAM1 (↓)	(68)
14 Obese	<50 g/d	19:70:11	42:23:35	250–500 mg DHA	6 mo	Baseline	Yes		↓	↓	↓	↓	=	↓		(69)
28 Obese	<50 g/d	20:60:20			12 mo	Baseline	Yes		=	=	↓	↓				(70)
11 Obese with T2D		20:35:45			24 wk	Control			↓	↓	↓		↓	↓		(71)
10 Obese		20:25:55			8 wk	Baseline			↓			=				(21)
15 Firefighters		23:29:48			4 wk	Baseline	Yes		↓	=	=	=				(72)
30 Type 2 diabetics		25:26:49	43:40:17		6 mo	Baseline	Yes		↓			=	=		IL1Ra (↓)	(73)
43 Severely obese	<30 g/d	32:25:43			6 mo	Baseline	Yes		↓		↓	=				(74)
75 Obese	<40 g/d	34:24:41	37:41:23		12 mo	Low fat	Yes		↓				=	=	IL8 (=), ICAM1(↓)	(75)
262 Diabetics					12 mo	Baseline		0.31	↓			↓			WBC (↓)	(22)
11 Multiple sclerosis subjects	<50 g/d CHO, >160 g fat				6 mo	Control		1.4	↓	=	=				ALOX5 mRNA (↓)	(76)
22 Healthy, obese women	<20-60 g/d				3 mo	Baseline	Yes		↓			↓				(77)
6 Overweight divers	<40 g/d				7 d	Baseline	Yes	0.9	↓					↓	Hyperoxic-induced IL6 (↓), IL1β (↓)	(78)

*The up arrow indicates “increased” and the down arrow indicates “decreased,” while = indicates “no change”*.

Despite the often-increased intake of saturated fats, many studies evaluating the effect of CHO restrictive diets on inflammatory markers suggest that these diets reduce or have no effect on inflammation (Table 1). An important caveat in these studies, however, is that C-reactive protein (CRP) and IL-6, inflammatory markers commonly used to assess inflammatory status in these studies, are positively correlated with obesity (81). Since low CHO diets often inadvertently lead to lower calorie intake, which leads to the observed weight loss in these studies (Table 1) (67, 68, 82), it is possible that the observed reduction in CRP is attributable to diet-induced weight loss rather than the diet components themselves (60, 77, 83). As well, while Forsythe et al. (65) have demonstrated that increased saturated fat consumption does not translate to increased circulating saturated fat, the gastrointestinal (GI) tract remains exposed to a large quantity of fat in a low CHO, high fat/ketogenic diet. Increased fat intake may stimulate bile acid secretion, required to emulsify fat and promote intestinal absorption, and this bile acid may then be recycled in the liver to generate a secondary bile acid that, in turn, may promote colon cancer formation (84, 85). It is also possible that immune cells within our intestinal lining become activated by saturated fats through direct activation of Toll-like receptors (ie. TLR 2 and 4) (86), leading to intestinal inflammation. Further studies evaluating the long-term effects of a low CHO diet that incorporates different types of fatty acids are therefore warranted.

## Ketone Bodies and Cancer

One of the main consequences of consuming a ketogenic diet is an increase in circulating ketone bodies, β-hydroxybutyrate (β-HB), acetoacetate and acetone. As mentioned earlier, employing a ketogenic diet to prevent or treat cancer is particularly appealing because of the possibility that many cancer cells are unable to use ketone bodies as an energy source. However, since ketogenic diets are hard to adhere to, the use of ketone supplements is currently being evaluated as a way to reach ketosis in the absence of a ketogenic diet. This is being pursued on the assumption that ketone bodies themselves, rather than the lower levels of blood glucose and insulin associated with low CHO, ketogenic diets, play a critical role in the reduction in tumors seen with ketogenic diets (28, 87). Of the three ketone bodies, β-HB, has been reported to be uniquely anti-inflammatory (88), in part because it suppresses IL-1β expression in bone marrow derived macrophages (89), and promotes uncoupling protein 2 expression in mitochondria (90). The latter observation might account for its reported ability to lower oxidative stress (91). In addition, β-HB has been shown to inhibit HDAC activity and this may contribute to its protective effects against oxidative stress (91, 92) and thus extend lifespan (93).

## The Case for Soluble Fiber and Resistant Starch

**Figure 1:**
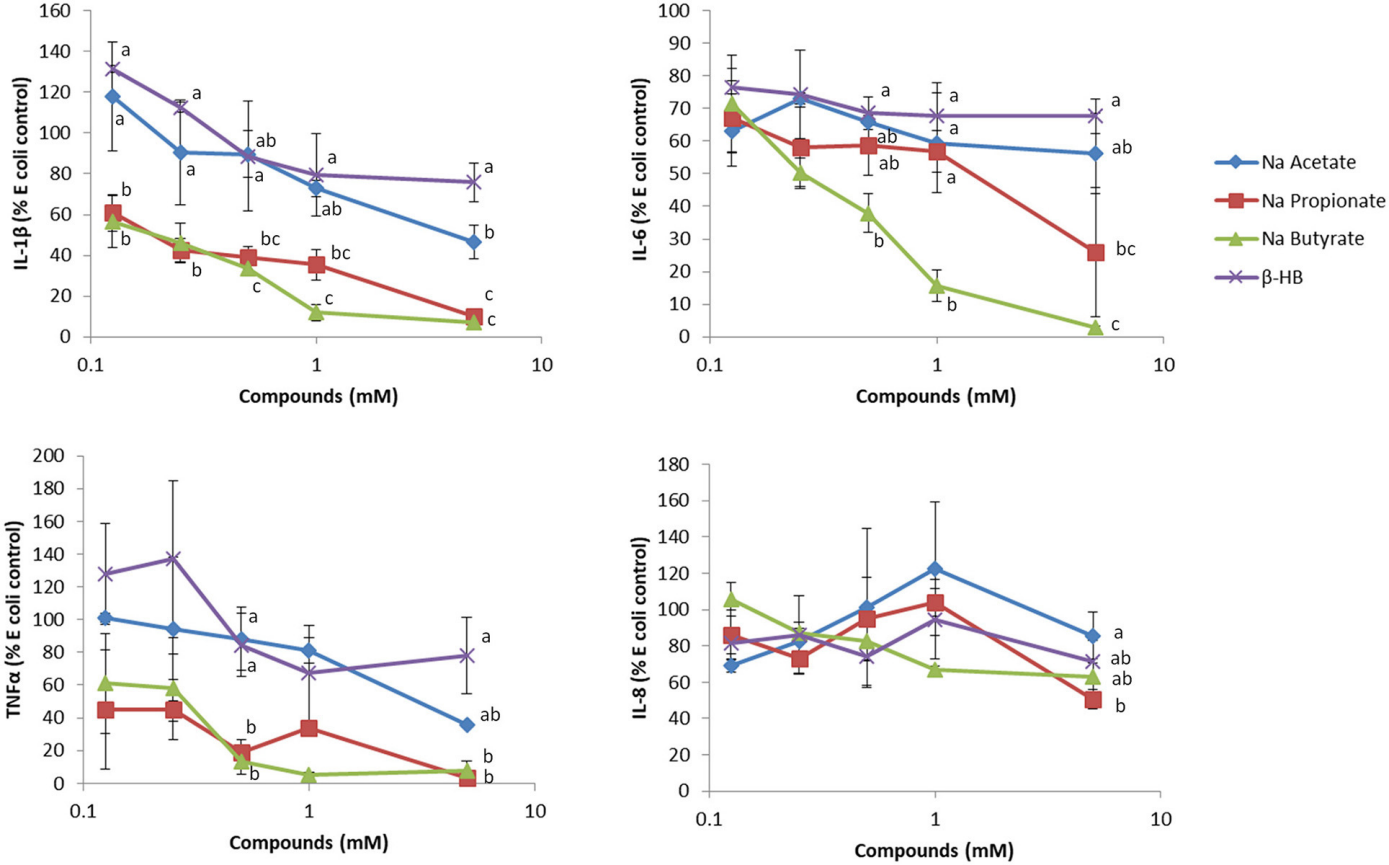
The effect of sodium acetate, propionate, butyrate and β-hydroxybutyrate (β-HB) on *E. coli*-stimulated inflammatory cytokines from whole blood. Briefly, varying concentrations of SCFAs or β-HB dissolved in PBS were added to 50 μL whole blood, collected in sodium heparin glass tubes, for 15 min at 37°C, in a low (5%) oxygen incubator, and then challenged with *Escherichia coli* (One Shot INV 110, Life Technologies, Burlington, ON) at a final concentration of 2 × 104 cells/mL for 7 h as previously described (94). PBS (100 μL) was added to the whole blood, mixed well and upon centrifugation at 424 × g at 4°C for 5 min in an Allegra X-12R centrifuge, the plasma was recovered for ELISA analysis (*n* = 3). This experiment was repeated two additional times, and results shown are representative of three independent experiments. Results are expressed as percentages, relative to *E.coli*-stimulated cells. The effect of the sodium acetate, butyrate, propionate and β-HB at each concentration on the cytokine/chemokine level was evaluated using one way ANOVA, followed by Tukey's multiple comparison *post-hoc* analysis. The different letters denote significant (*P* < 0.05) differences between treatments at the same concentration. Experiments using blood collected from human subjects were reviewed and approved by the joint Clinical Research Ethics Board of the University of British Columbia and the BC Cancer (#H12–00727).

While βHB has been reported to have anti-inflammatory effects, we found that βHB at a level comparable to that typically found in nutritional ketosis is very weak in terms of reducing inflammation in human whole blood assays. In these *ex-vivo* studies, we challenged whole blood with *E. coli* bacteria. While *E. coli* stimulated a robust secretion of IL-6, IL-8, TNF-α and IL-1β, β-HB had minimal effects in reducing this secretion (Figure 1). Since β-HB levels in blood during nutritional ketosis range from 0.5 to 3 mM (95), we believe that our assay results are physiologically relevant.

On the other hand, when we tested the short chain fatty acids (SCFAs), i.e., acetate, propionate and butyrate, typically generated during fermentation of soluble fiber and resistant starch in the large intestine, we found far more pronounced anti-inflammatory effects on blood samples. We tested these short chain fatty acids at concentrations typically found in peripheral and portal blood [~0.1–0.4 mM (96)]. In the colon where SCFAs are produced by the gut microbiome, SCFAs can reach 150 mM (97). Of the SCFAs tested, butyrate, the main source of energy for colonocytes (98), was the most anti-inflammatory, which is interesting since it has been reported to trigger apoptosis in colon cancer cells, therefore eliciting protection against colon cancer formation. The chemopreventive effect of butyrate is often attributed to its ability to inhibit HDAC activity, which in turn regulates gene expression and anti-inflammatory activity (99). Since low CHO diets are often low in both soluble fiber and resistant CHOs, which, as mentioned above, are precursors to gut-microbiome generated SCFAs, we suggest that their consumption, either by eating fruits and vegetables or by supplementation of low CHO diets, should be encouraged.

## Carbohydrate Restriction in Human Studies

Because low CHO and ketogenic diets have been considered controversial, there have been very few human studies investigating the efficacy of CHO restriction for the treatment or prevention of cancer. There have been a few pilot or case studies with a small number of subjects, but a larger randomized controlled trial still needs to be performed. In one of these pilot studies, adherence was very poor, highlighting another potential roadblock to studies aiming to evaluate the efficacy of CHO-restricted diets in cancer treatment (100). There are, however, a growing number of reports in the literature on the effect of low CHO diets on metabolic profiles (Table 1). In these studies, low CHO diets, while not always effective in lowering blood glucose, were often reported to lower circulating insulin levels and other inflammatory markers such as CRP. When ketogenic diets were tested, increased levels of ketone bodies, such as β-HB, were found. These are favorable changes that may work independently or together to reduce cancer risk (Table 1). With the increasing evidence of benefit in pre-clinical studies, we hope that more high quality studies will address the potential usefulness of low CHO diets in cancer.

## Discussion

While pre-clinical studies evaluating the safety and efficacy of low CHO diets in cancer prevention or treatment show promise, more studies are required to ensure the safety and efficacy of these diets in humans. Specifically, there is a need to evaluate the effect of different fatty acids in low CHO and ketogenic diets on inflammatory status, since this may help formulate a more optimal diet plan that not only facilitates weight loss, but also acts to reduce inflammation. In addition, the long-term safety of low CHO diets is an important question that has not been sufficiently addressed. While many clinical trials demonstrate that increased saturated fat intake in the context of low CHO diets does not lead to increased inflammation, these studies have been short-term. Low CHO diets high in saturated fats tend to include a substantial proportion of meat, and the long-term consumption of meat may pose health consequences that may not be detected in short-term studies. The effect of high meat consumption in low CHO diets, in particular, may increase cancer risk, especially colon cancer. It is also possible that an increase in meat consumption may lead to an increase in heterocyclic aromatic amines and polyaromatic hydrocarbons, which are carcinogens typically found in cooked meat or fish (101, 102). In addition, soluble fiber and resistant starch intake might be severely compromised, which would result in low CHO followers losing out on the health benefits potentially derived from them. It will thus be of interest to determine if low CHO, high fat diets are associated with increased GI cancers if adopted long-term. In conclusion, it is evident from this perspective that further studies investigating the potential efficacy of low CHO diets in the prevention and treatment of cancer are both needed and warranted.

## Data Availability Statement

The original contributions generated in the study are included in the article/supplementary material, further inquiries can be directed to the corresponding author.

## Ethics Statement

The studies involving human participants were reviewed and approved by the Clinical Research Ethics Board of the University of British Columbia and BC Cancer (#H12-00727). The patients/participants provided their written informed consent to participate in this study.

## Author Contributions

IE wrote the first draft. GK edited, revised, and approved the manuscript. Both authors contributed to the article and approved the submitted version.

## Conflict of Interest

The authors declare that the research was conducted in the absence of any commercial or financial relationships that could be construed as a potential conflict of interest.
